# Spectrum of Systemic Auto-Inflammatory Diseases in India: A Multi-Centric Experience

**DOI:** 10.3389/fimmu.2021.630691

**Published:** 2021-03-19

**Authors:** Deepti Suri, Amit Rawat, Ankur Kumar Jindal, Pandiarajan Vignesh, Anju Gupta, Rakesh Kumar Pilania, Vibhu Joshi, Kanika Arora, Rajni Kumrah, Gummadi Anjani, Amita Aggarwal, Shubha Phadke, Fouzia N. Aboobacker, Biju George, Eunice Sindhuvi Edison, Mukesh Desai, Prasad Taur, Vijaya Gowri, Ambreen Abdulwahab Pandrowala, Sagar Bhattad, Swati Kanakia, Marco Gottorno, Isabella Ceccherini, Adriana Almeida de Jesus, Raphaela Goldbach-Mansky, Michael S. Hershfield, Surjit Singh

**Affiliations:** ^1^Post Graduate Institute of Medical Education and Research (PGIMER), Chandigarh, India; ^2^Sanjay Gandhi Post Graduate Institute of Medical Sciences (SGPGI), Lucknow, India; ^3^Christian Medical College and Hospital, Vellore, India; ^4^Bai Jerbai Wadia Hospital for Children, Mumbai, India; ^5^Aster Cauvery Medical Institute Hospital, Bengaluru, India; ^6^Lilavati Hospital and Research Center, Mumbai, India; ^7^Center for Autoinflammatory Diseases and Immunodeficiency, Istituto di Ricovero e Cura a Carattere Scientifico Instituto Giannina Gaslini, Genoa, Italy; ^8^Translational Autoinflammatory Diseases Section, Laboratory of Clinical Immunology and Microbiology, National Institute of Allergy and Infectious Diseases, National Institutes of Health, Bethesda, MD, United States; ^9^Duke University Medical Centre, Durham, NC, United States

**Keywords:** systemic autoinflamatory diseases, India, deficiency of adenosine deaminase 2, NOMID/CINCA, hyper IgD syndrome, A20 (TNFAIP3), inflammasome, Type I interferonopathies

## Abstract

**Background:** Systemic autoinflammatory diseases (SAID) are rare inherited disorders involving genes regulating innate immune signaling and are characterized by periodic or chronic multi-systemic inflammation.

**Objective:** To describe spectrum of clinical, immunological, molecular features, and outcomes of patients with SAID in India.

**Methods:** Request to share data was sent to multiple centers in India that are involved in care and management of patients with Inborn Errors of Immunity. Six centers provided requisite data that were compiled and analyzed.

**Results:** Data on 107 patients with SAID were collated—of these, 29 patients were excluded due to unavailability of complete information. Twelve patients (15%) had type 1 interferonopathies, 21 (26%) had diseases affecting inflammasomes, 30 patients (41%) had non-inflammasome related conditions and 1five patients (19%) had Periodic Fever, Aphthous Stomatitis, Pharyngitis, Adenitis (PFAPA). Type1 interferonopathies identified in the cohort included patients with Deficiency of Adenosine Deaminase 2 (*DADA2)* (six patients; five families); STING-associated vasculopathy infantile-onset (SAVI) (three patients, one family); Spondyloenchondro-dysplasia with Immune Dysregulation (SPENCD) (two patients). Diseases affecting inflammasomes include Mevalonate Kinase Deficiency (eight patients); Cryopyrin-Associated Periodic Syndromes (CAPS) (seven patients); NLR Family, Pyrin domain-containing 12 **(**NLRP12) (two patients); Familial Mediterranean fever (FMF) (two patients); Autoinflammation and PLCG_2_-associated antibody deficiency and immune dysregulation (APLAID) (two patients). TNF receptor-associated periodic syndrome (TRAPS) (three patients); A20 haploinsufficiency (four patients); Deficiency of Interleukin 1 Receptor Antagonist (DIRA) (two patients) were categorized as non-inflammasome related conditions. There were significant delays in diagnosis Corticosteroids and other immunosuppressive agents were used for treatment as anti-IL-1 drugs and other biological agents were and still are not available in India. Eight (16.3%) patients had so far succumbed to their illness.

**Conclusions:** This is the first nationwide cohort of patients with SAID from India. Clinical manifestations were diverse. Overlapping of clinical features with other relatively common rheumatological disorders often resulted in delays in diagnosis. More nationwide efforts are needed to enhance awareness of SAID among health care professionals and there is an urgent need to make targeted immunotherapies universally available.

## Introduction

Systemic autoinflammatory diseases (SAID) are complex inherited disorders caused by defects in several genes regulating innate immune signaling and are characterized by periodic or chronic multisystem sterile inflammation (1–3).

The term “autoinflammatory disorders” was coined in 1999 by Daniel Kastner's group when they proposed a new group of immunological diseases (4). The paper described genetic background of familial Hibernian fever, and rechristened it as “TNF receptor-associated periodic syndrome (TRAPS).” It also linked it with previously described mutations in Pyrin (*MEFV)*gene that causes familial Mediterranean fever (FMF) (4–6). In 2010, Kastner et al. defined autoinflammatory diseases as “clinical disorders marked by abnormally increased inflammation, mediated predominantly by cells and molecules of the innate immune system with a significant host predisposition” (1, 7). Euro fever registry and Pediatric Rheumatology International Trials Organization (PRINTO) have also proposed classification criteria for different hereditary recurrent fever syndromes (8).

SAIDs can be monogenic and polygenic or multifactorial (9, 10). Monogenic SAID (e.g., TRAPS, FMF) follow Mendelian inheritance and result from pathogenic variants in a single gene. On the other hand, disorders such as systemic juvenile idiopathic arthritis, Periodic Fever, Aphthous Stomatitis, Pharyngitis, Adenitis (PFAPA) syndrome and Adult-Onset Still Disease have polygenic or multifactorial etiology. The 2019 International Union of Immunological Societies (IUIS) Expert Committee classified monogenic SAID into 3 major groups: Type 1 interferonopathies, defects affecting the inflammasome and non-inflammasome-related conditions (11).

Over the last 2 decades due to an increasing awareness and availability of high throughput genetic sequencing techniques, there has been an exponential increase in discovery of genes responsible for SAID (2, 12, 13). Further, molecular insights of these disorders have provided the basis for new therapeutic interventions leading to improved outcomes and long-term survivals. There is paucity of data on SAID from India with published literature comprising of only anecdotal case reports (14–21). In this manuscript we describe clinical features, molecular profile, treatment and outcome in patients with monogenic SAID from six centers in our country. This paper reports nationwide cohort on SAID.

## Patients and Methods

Centers supported by the Foundation for Primary Immunodeficiency Diseases (FPID), USA, and other institutions involved in care of patients with Inborn Errors of Immunity (IEI)across India were contacted to share details of patients with SAID on a template designed by lead author (DS). Data including demographics, prominent clinical manifestations, laboratory investigations, molecular results, treatment regimens, and long-term outcomes were collated on predesigned Microsoft Excel sheet. Findings of radiology and histopathology were also recorded. Participating centers included Postgraduate Institute of Medical Education and Research (PGIMER), Chandigarh, North India (52 patients); Sanjay Gandhi Postgraduate Institute of Medical Sciences (SGPGIMS), Lucknow, North India (25 patients); Christian Medical College, Vellore (12 patients), Bai Jerbai Wadia Hospital for Children (BJWHC), Mumbai, West India (10 patients); and ASTER CMI Hospitals, Bengaluru, South India (seven patients), Lilavati Hospital and Research Center,Mumbai, West India (one patient).

## Definition of Said

Several definitions have been proposed for SAID (4, 8, 11, 22). For the purpose of this study we have used European Society for Immunodeficiencies (ESID) working group definition for the categorization of SAID. ESID has defined “unclassified autoinflammatory diseases” to be characterized by recurrent fever (temperature >38°C) having occurred on at least six occasions with exclusion of other known infective/inflammatory autoimmune disorders and documented evidence of increased inflammatory markers [erythrocyte sedimentation rate (ESR), C-reactive protein (CRP)], age of onset under 40 years and predominantly but not exclusively with systemic symptoms (23). In the present study all patients who fulfilled ESID working group definition and had molecular confirmation of monogenic SAID were included. Patients with polygenic SAID (e.g., systemic juvenile idiopathic arthritis, chronic non-infectious osteitis) and infantile inflammatory bowel disease were excluded.

All patients were further classified into three subtypes according to 2019 IUIS classification for SAID (11). Coatamer complex one protein alpha subunit (COPA) syndrome was classified as Type 1 interferonopathy (24, 25). Patients without molecular confirmation of diagnosis and/or could not be classified in accordance with IUIS classification were also excluded. Some patients included in this series have been reported earlier and these have been duly cited (14, 17, 19, 26, 27).

## Molecular Investigations

Molecular analysis of patients for PGIMER, Chandigarh was performed at Pediatric Allergy Immunology Laboratory at PGIMER, Chandigarh or in collaboration with international centers, namely Center for Autoinflammatory Diseases and Immunodeficiency, Genoa, Italy (1three patients) and National Institutes of Health (NIH), USA (three patients). Measurement of plasma adenosine deaminase 2 (ADA2) activity in extracts of dried plasma spots was performed in the laboratory of Dr. Michael Hershfield at Duke University School of Medicine, Durham NC, USA (28).

## Laboratory Investigation at PGIMER, Chandigarh

Molecular analysis of Nucleotide binding oligomerization domain 2 (*NOD* 2) gene in patients suspected to have Blau syndrome (11/14 patients) and Adenosine deaminase 2(*ADA2)* gene for Adenosine Deaminase 2 (ADA2) deficiency was performed in-house in Pediatric Immunology Laboratory, Advanced Pediatric Center by Sanger sequencing. Exon-4 of *NOD2* gene was amplified using specified oligonucleotide primers and results were analyzed using Codon Code Aligner software (Codon Code Corporation, Massachusetts, USA). Screening of hotspot region (exon 2) of *ADA*2 gene was also performed in patients clinically suspected to have Deficiency of Adenosine Deaminase 2 (DADA2).

Molecular analysis in most patients at other centers was carried out at commercial laboratories that use targeted gene panel by Next Generation Sequencing (NGS) techniques. Sanger sequencing was used to confirm the variants obtained by NGS.

## Results

**Figure 1:**
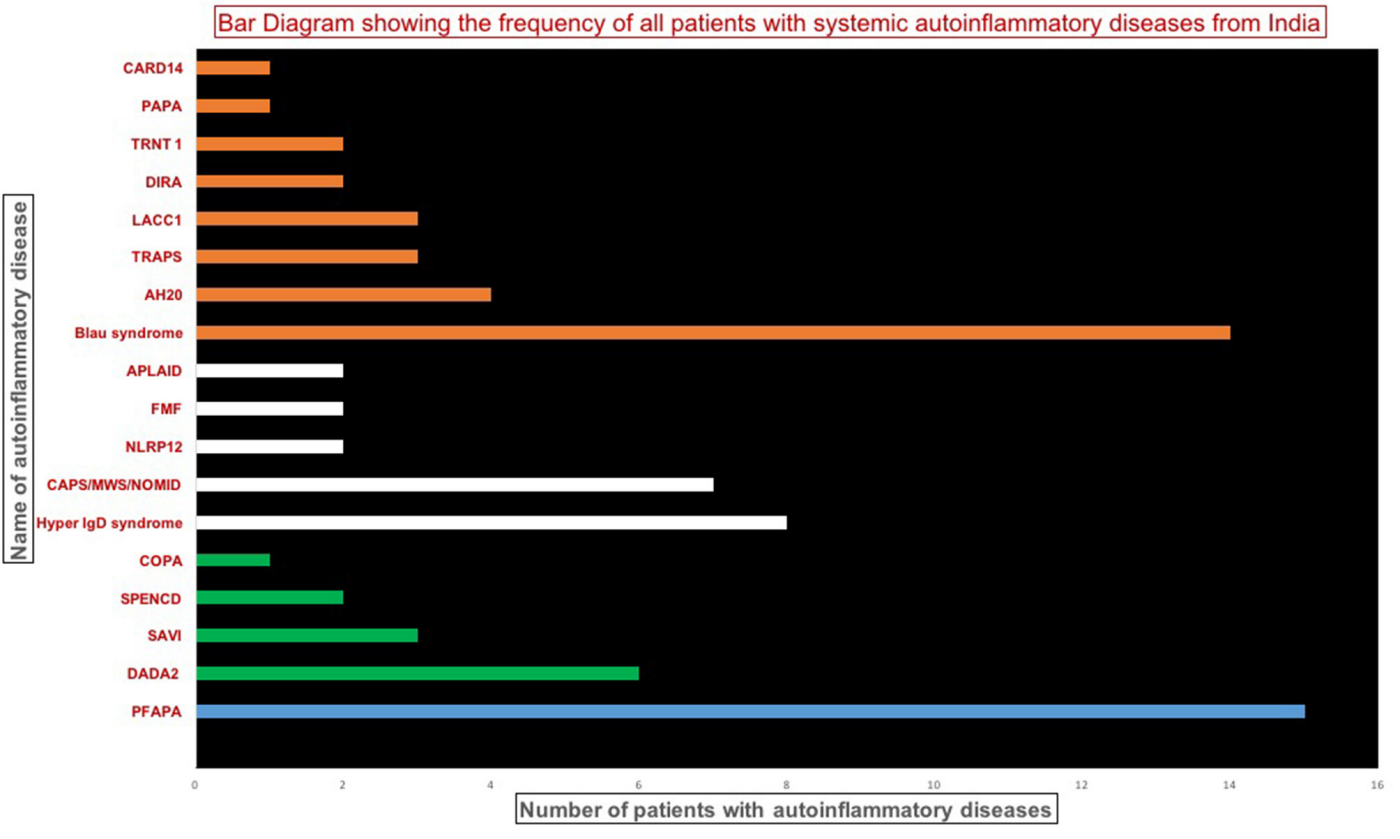
Bar diagram showing the frequency of patients with various systemic autoinflammatory disease. DADA2, Deficiency of adenosine deaminase 2; SAVI, STING-associated vasculopathy infantile-onset; SPENCD, Spondyloenchondro-dysplasia with Immune Dysregulation; CAPS, Cryopyrin-Associated Periodic Syndromes; NLRP12, NLR Family, Pyrin domain-containing 12; FMF, Familial Mediterranean fever; APLAID, Autoinflammation and PLCG2-associated antibody deficiency and immune dysregulation; TRAPS, TNF receptor-associated periodic syndrome; HA20, A20 haploinsufficiency; LACC1, Laccase Domain Containing 1; DIRA, Deficiency of Interleukin 1 Receptor Antagonist; TRNT1, TRNA nucleotidyl transferase; PAPA, Pyogenic Arthritis; Pyoderma gangrenosum and Acne; COPA, Coatamer complex 1 protein alpha subunit (COPA) syndrome; CARD14, Caspase Recruitment Domain Family Member 14.

Data on 107 patients with SAID were collated from various centers in India. Of these, 19 patients had to be excluded as molecular confirmation was not available. Ten patients with variants of unknown significance (VUS) in genes associated with SAIDs were also excluded if found inconsistent with clinical profiles or non-pathogenic based on predictive analysis tools. Remaining 78 patients (Figure 1) included Periodic Fever, Aphthous Stomatitis, Pharyngitis, Adenitis (PFAPA) (15 patients (19%) from PGIMER); type 1 interferonopathies (1two patients, 15%); diseases affecting inflammasomes (21 patients, 26%); and non-inflammasome related conditions (30 patients, 38%%). Clinical details of patients with PFAPA (1five patients) and Blau syndrome (14 patients) are not being presented in the current manuscript (Suri et al., manuscript in submission).

## Clinical Profile Of Patients With Type 1 Interferonopathies

Type 1 interferonopathies identified in the cohort included patients with DADA2 (six patients; five families); STING-associated vasculopathy infantile-onset (SAVI) (three patients from one family); Spondylo enchondro dysplasia with Immune Dysregulation (SPENCD) (two patients) and Coatamer complex one protein alpha subunit (COPA syndrome) (one patient) (Table 1).

**Table 1:** Clinical manifestations, molecular profile, treatment, and outcome of patients with type I interferonopathies (*n* = 12).

**Center**	**Patient (Age at diagnosis/Sex)**	**Age of onset of symptoms**	**Clinical features**	**Laboratory features**	**Family history (Consanguinity/siblings affected)**	**Initial Diagnosis**	**Molecular details**	**Treatment details**	**Follow-up duration and outcomes**
**Deficiency of Adenosine Deaminase 2 (DADA2) (*****n=*** **6)**									
PGIMER	Pt. 1 (31y/M)	3.3y	• Fever • Rash • Hypertension • Recurrent strokes at 3 and 16 years of age	CRP: 32 mg/L ESR: 20 mm/h MRI brain: multiple infarcts right MCA territory and right posterior circulation CTA: microaneurysms in branches renal artery Muscle biopsy: healed arteritis	Third degree consanguinity	PAN	*ADA2* exon 2; c.140G>T; p.Gly47Val Homozygous; missense Previously reported: Yes	CS, AZR, enalapril, aspirin Change in treatment after diagnosis: Aspirin stopped, HCQs added and planned for ant-TNF	34 years and doing well
	Pt. 2 (13 y/F)	5 y	• Fever • Recurrent abdominal pain • Hypertension • Optic atrophy • Left hemiparesis and facial palsy intestinal perforation	CRP: 45 mg/L ESR: 40 mm/h DSA: multiple microaneurysms involving bilateral interlobar and segmental branches of renal artery, branches of gastroduodenal artery, distal branches of SMA and IMA GI Biopsy: Ulcer, ischemic, gangrene, perforation in ileum. Chronic inflammation in recto-sigmoid junction Plasma ADA2 activity: 1.1 mU/g protein mL Plasma ADA2 activity (Father): 42.5 mU/g protein mL Plasma ADA2 activity (Mother): 69.5 mU/g protein mL	Sister of Pt. 3	PAN	*ADA2* exon 2; c.139G>C; p.Gly47Arg Homozygous missense Previously reported: Yes	CS, CYC (10 pulses), AZR, aspirin Change in treatment after diagnosis: Aspirin stopped, HCQs added and planned for anti-TNF	8 year and doing well
	Pt.3 (18 y/M)	17 y	• Sudden Painless loss of vision • Raynaud phenomenon, CRAO	CRP: 10 mg/L ESR: 12 mm/h CTA: Normal study Plasma ADA2 activity: 0.3 mU/g protein mL	Brother of Pt. 2	PAN	*ADA2* exon 2; c.139G>C; p.Gly47Arg Same as the sibling (Pt. 2)	CS, CYC (6 pulses), AZR, aspirin, LMWH Change in treatment after diagnosis: Aspirin stopped, HCQs added and planned for anti-TNF	3 years and doing well
SGPGI	Pt. 4 (17 y/M) (29)	5 y	• Fever • Vasculitic ulcers • Seizures, • Recurrent stroke with neurological deficits • VI^th^ Cranial Nerve palsy, median nerve neuropathy, • GI bleed	Skin Biopsy: Necrotizing cutaneous vasculitis	No	PAN	*ADA2* exon 2; c.139G>C; p.Gly47Arg; exon 2; c.278T>C; p.Ile93Thr Previously reported: Yes Homozygous missense variation	CS, AZR Change in treatment: anti-TNF commenced	1 year and doing well
	Pt. 5 (48 y/M) (29)	8 y	• Fever • Ulcers and rash • Recurrent stroke along with neurological deficits, mononeuritis multiplex, CRAO	CRP: 5.11 mg/L ESR: 30 mm/h C3/C4: 129/32.3		PAN	*ADA2* exon 2; c.139G>C; p.Gly47Arg Homozygous missense variation Previously reported: Yes	CS, MMF Change in treatment: Stopped aspirin	doing well
Aster CMI	Pt. 6 (0.9 y/F) (29)	5 months	• Fever • Anemia • generalized lymphadenopathy, splenomegaly	CRP: 102 mg/L ESR: 155 mm/h Bone marrow: Normocellular bone marrow with trilineage hematopoiesis IgG: 1,640 mg/dL IgA: 101 mg/dL IgM: 96 mg/dL IgE: 3.7 mg/dL	No	-	*ADA2* exon 2; c.139G>C; p.Gly47Arg Homozygous missense variation Previously reported: Yes	Injection etanercept	Doing well
**STING-associated vasculopathy with onset in infancy (SAVI) (*****n=*** **3)**									
PGIMER	Pt 7 (10 y/F) (19)	0.91 y	• Fever, • Failure to thrive, • Deforming inflammatory arthritis with contractures of small and large joints • ILD, corneal • Opacity in right eye	CRP: 97.23 mg/L ESR: 120 mm/h CT chest: ILD RA factor: positive ANA: 4+ RIM IgG: >2,535 (540–1,610) IgA: 436 (70–250) C3: 166 mg/dl (89–187) C4: 20 mg/dl (16–38) Anti ds-DNA: 10.8 IU/ml (<25- Negative) Serum IL-6: 3,700 pg/ml Serum IL-10: 13,900 pg/ml Interferon levels elevated	Brother and Father affected (Pt. 8 and Pt. 9)	JIA, COPA	*TMEM173* exon5; c.463G>A; p.Val155Met heterozygous missense variation Previously reported: Yes	CS, MTX, AZR, Naproxen, HCQ	Alive
	Pt 8 (3 y/M) (19)	2 y	• Fever • Polyarthritis (bilateral knee, small joints of the hands) • Rash ILD	CRP: 12.98 mg/L ESR: 108 mm/h CT chest: ILD RA factor: negative ANA: 2+ Speckled IL-6: 3,500 pg/ml IL-10: 14123 pg/ml Interferon levels elevated	B/o Pt. 7	JIA, COPA	*TMEM173* exon5; c.463G>A; p.Val155Met heterozygous missense variation Same as Pt. 7	AZR, MTX	Well
	Pt 9 (4y/-) (19)		• Deforming inflammatory polyarthritis involving small and large joints • ILD • Peripheral vascular disease of bilateral lower limbs with guillotine amputation of right midfoot and 2^nd^ toe in the year 2008	ESR: 12 mm/h RA Factor: 6.49 mg/L ANA (IF): 3 + diffuse CT chest: Emphysematous changes and interstitial thickening in bilateral lungs consistent with ILD	F/o Pt. 7	RA	*TMEM173* exon5; c.463G>A; p.Val155Met heterozygous missense variation Same as Pt. 7	-	-
**Spondyloenchondrodysplasia (SPENCD) (*****n=*** **2)**									
SGPGI	Pt. 10 (15 y/F)	13 y	• Fever • Seizure • Stroke • Optic atrophy • Hypertensive • Short stature	MR brain: Basal ganglion calcification Renal biopsy: IgA nephropathy ANA–Positive Anti dsDNA: 67.5 IU C3/C4: 105 mg/dL/29.6 mg/dL IgG: 3,590 mg/dl IgA: 621 mg/dl IgM: 60.9 mg/dl	No	SLE	*ACP 5* exon 3; c.550C>T; p.Gln184* exon 4; c.740T>G; p.Leu247Arg	HCQs, antihypertensive drugs	NA
Lilavati Hospital	Pt. 11 (4y/F)	1y	• Fever • Bleeding (Skin, mucosal and intracranial) • Anemia • Facial dysmorphism (delay in motor and cognitive milestones, fronto-parietal bossing, hyperteleorism, low set ears	X-ray wrist: metaphyseal dysplasia CT brain: Symmetrical bilateral basal ganglion calcifications and gliotic area noted in left Parieto- Temporal area Bone marrow biopsy: hypercellular marrow with erythroid and megakaryocytic hyperplasia. Increased bone marrow fibrosis DCT ICT: strongly positive multiple antibodies Cold agglutinin: positive	No	Early onset Immune thrombocytopenia	*ACP 5* exon 1; c.136delc; p.R46Gfs*24 Homozygous nucleotide deletion Parents heterozygous for the same variant	Multiple packed cell transfusions and platelet transfusions IVIg, CS, dapsone, cyclosporine	Doing well
**Coatomer protein complex subunit alpha (COPA) defect (*****n=*** **1)**									
PGIMER	Pt. 12 (11 y/M) (30)	5 Y	Polyarthritis, ILD	CRP: 32 mg/L ESR: 23 mm/h HRCT: ILD IgG: 1,453 mg/dL IgA: 131 mg/dL IgM: 135 mg/dL RA, CCP: Positive ANA: 3+ speckled ANCA: negative	Father died due to progressive lung disease	Poly JIA	*COPA* exon 9 (intron 9-10 junction) c.841C>T, p.Arg281Trp Novel heterozygous splice-site mutation Sangers (PGI)	CS, MTX, HCQs	Alive

*ACP5, Acid phosphatase 5; ANCA, Anti neutrophil cytoplasmic antibody; ADA2, Adenosine deaminase 2; ANA, Antinuclear antibodies; anti TNF, Tumor necrosis factor; Aster CMI, Aster CMI Hospital, Bengaluru, India; AZR, Azathioprine; BJWHC, Bai Jerbai Wadia Hospital for Children, Mumbai, India; CMC, Christian Medical College and Hospital, Vellore, India; CRAO, Central retinal artery occlusion; CRP, C-reactive protein; CS, Corticosteroids; CT, Computed tomography; CTA, Computed tomography angiography; CYC, intravenous pulse cyclophosphamide; DCT, Direct Coombs test; DSA, Digital subtraction angiography; ESR, Erythrocyte sedimentation rate; HCQS, Hydroxychloroquine; HRCT, High resolution computed tomography GI, Gastrointestinal; ILD, Interstitial lung disease; IMA, Inferior mesenteric artery; JIA, Juvenile idiopathic arthritis; Lilavati Hospital, Lilavati Hospital and Research Center, Mumbai, India; LMWH, Low molecular weight heparin; MCA, Middle cerebral artery; MRI, Magnetic resonance imaging; MTX, Methotrexate; MMF, Myocphenolate mofetil; PAN, Polyarteritis nodosa; PGIMER, Post Graduate Institute of Medical Education and Research (PGIMER), Chandigarh, India; RA, Rheumatoid arthritis; SGPGI, Sanjay Gandhi Post Graduate Institute of Medical Sciences (SGPGI), Lucknow, India; SLE, Systemic lupus erythematosus; SMA, Superior mesenteric artery; TMEM173, Transmembrane protein 173; Y, years*.

### DADA2

Age of onset of symptoms in patients with DADA2 ranged from 5 months to 17 years while age at diagnosis ranged from 9 months to 48 years. All patients with DADA 2 were diagnosed and managed as polyartritis nodosa (PAN). Family history was contributory in three patients (patient no. 2, 3, and 5). Predominant clinical features included fever (4/5), recurrent stroke (3/5), vasculitic rash (3/5), and retinal changes (2/5). Patient one had presented with hypertensive stroke at 3.3 years of aging 1992 and had second episode at the age of 16 years in 2002. The diagnosis of DADA2 was established in 2018 after three decades of follow-up.

**Figure 2:**
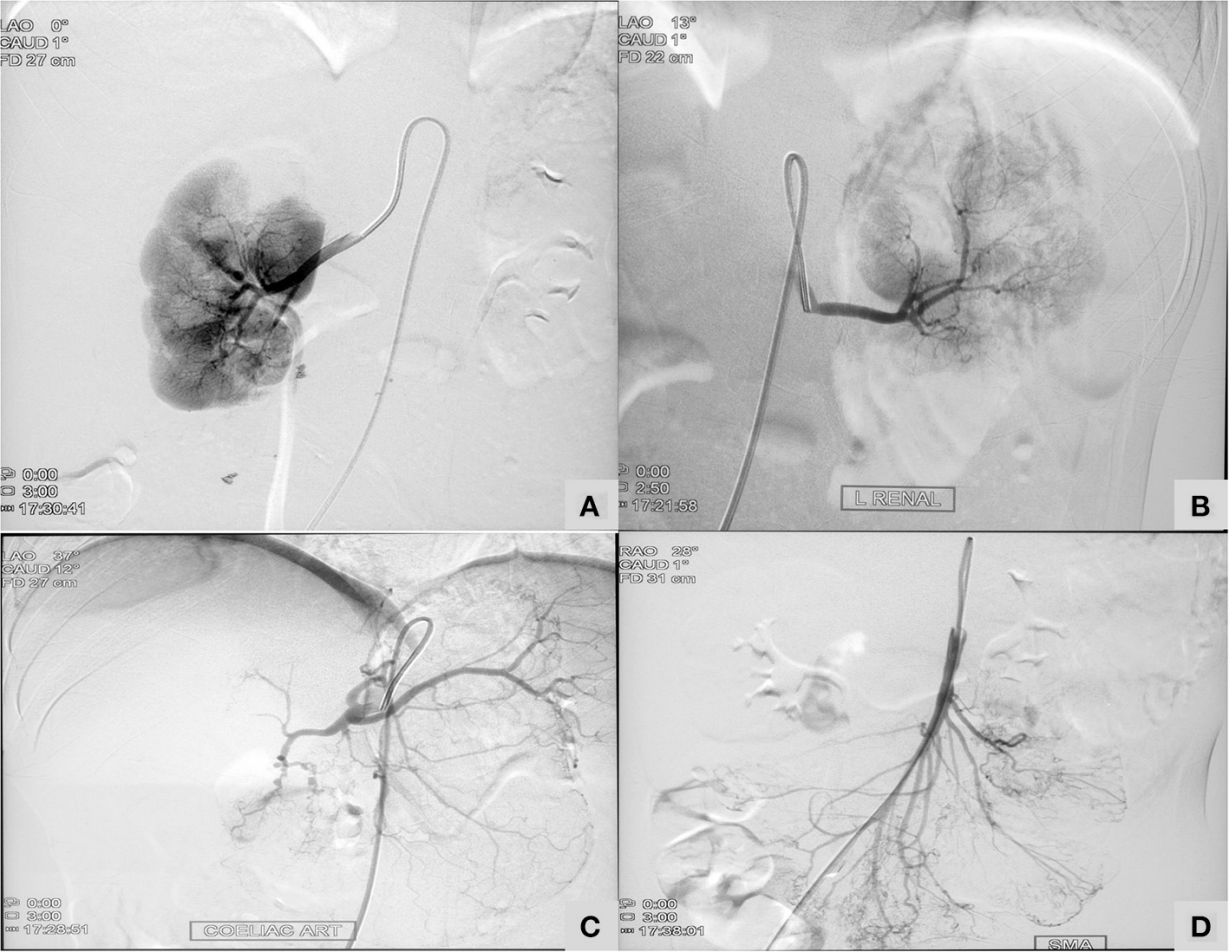
A 13-year-old girl with Deficiency of Adenosine Deaminase 2 (Table 1; Patient two) showing micronaeurysm on intervation catheter angiography in bilateral renal arteries **(A,B)**, celiac artery **(C)**, and superior mesenteric artery **(D)**.

Patient no 3 was diagnosed to have central retinal artery occlusion. Inflammatory markers were persistently normal. His sister (patient two) was under treatment and follow up for PAN. She had presented with recurrent abdominal pain with perforation peritonitis and catheter angiography had revealed microaneurysms in mesenteric arteries and renal arteries (Figure 2). In view of family historyDAD2 was suspected and mutation in *ADA2* gene was detected. Establishment of diagnosis lead to stoppage of aspirin and commencement of anti-TNF agents.

### SAVI

A 10-year-old girl (patient no. 7), previously reported (19) had presented with fever, polyarthritis, and interstitial lung disease (ILD). Initial diagnosis of juvenile idiopathic arthritis with ILD was considered. Younger sibling (patient no. eight) and father (patient no. nine) of index patient also had similar symptoms. Father gave history of gangrene of both lower limbs with amputation of right midfoot and left 2nd toe. Exome sequencing revealed pathogenic variant in Transmembrane protein 173 (*TMEM173)* gene confirming the diagnosis of SAVI.

### SPENCD

A 13-year-old girl (patient no 10) had persistent pyrexia, decreased vision with bilateral optic atrophy, hypertensive stroke, seizures, and proteinuria. Investigations showed hypergammaglobulinemia and positive antinuclear antibodies (ANA) with elevated anti-double stranded DNA (dsDNA) but normal complements. Initial diagnosis of systemic lupus erythematosus was proffered. Renal biopsy revealed IgA nephropathy. Magnetic Resonance Imaging (MRI) brain showed basal ganglia calcifications. Exome sequencing revealed pathogenic variant in Acid phosphatase 5 (*ACP 5*)gene which was confirmed on Sanger sequencing.

**Figure 3:**
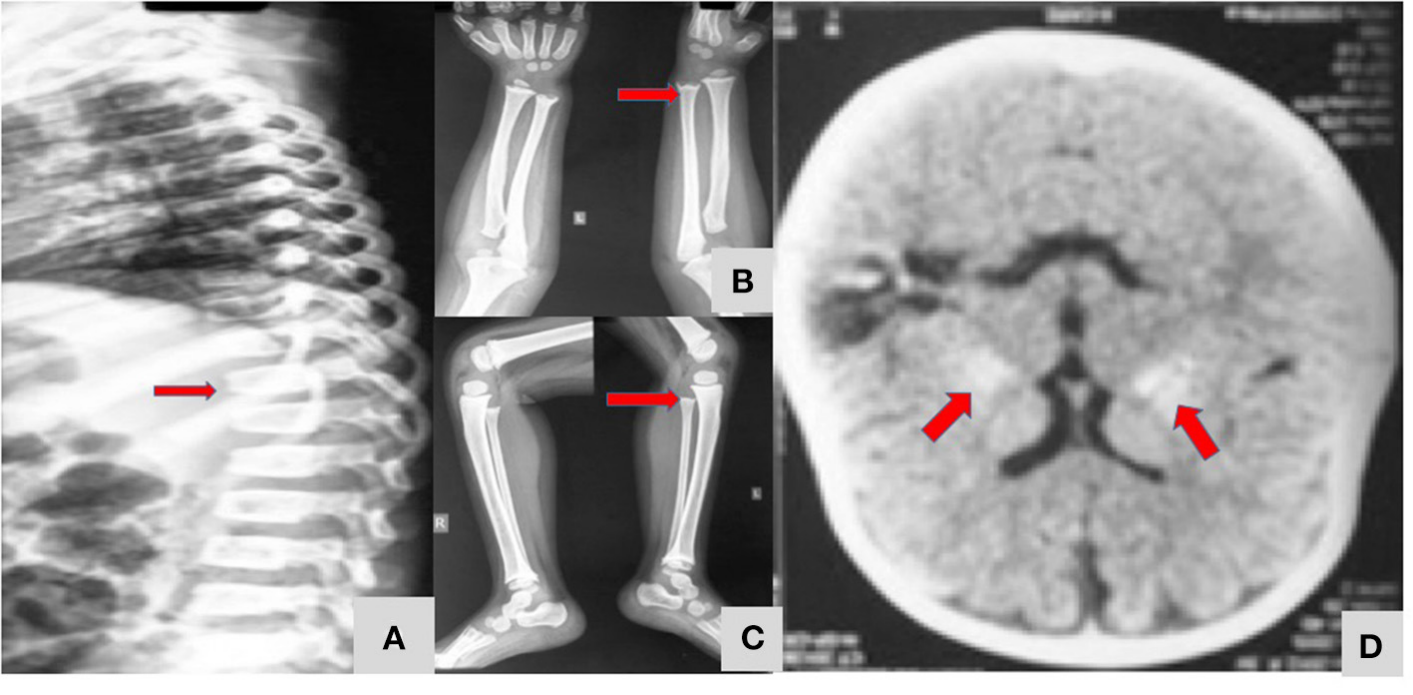
A 4 year-old-girl with Spondyloenchondrodysplasia (Table 1; Patient 11) showing fish mouth vertebra **(A)**, metaphyseal dysplasia of long bones of upper and lower limbs **(B,C)**, and bilateral symmetrical basal ganglia calcification **(D)**.

A 4-year-old girl (patient no 11) had presented with bleeding manifestations (skin, mucosal and intracranial bleed) since infancy (Figure 3). She had steroid refractory anemia and thrombocytopenia with no autoantibodies and hypocellular bone marrow. She was later noted to have short stature and metaphyseal dysplasia along with bilateral basal ganglia calcification. Targeted gene panel revealed homozygous nucleotide deletion in exon 1 of *ACP5* gene.

### COPA Syndrome

The index patient (patient no 12), previously reported (30) was diagnosed to have COPA syndrome when they had presented with rheumatoid factor positive deforming polyarthritis and interstitial lung disease. His father also had arthritis and had succumbed to progressive lung disease.

## Clinical Profile of Patients With Defects Affecting the Inflammasomes

Twenty-one patients were classified to have inflammasomopathies (*n* = 21, 26%). These included Mevalonate Kinase Deficiency (HyperIgD syndrome) (eight patients); Cryopyrin-Associated Periodic Syndromes (CAPS) (seven patients); NLR Family, Pyrin domain-containing 12 (NLRP12) (two patients); FMF (two patients); Autoinflammation and PLCG_2_-associated antibody deficiency and immune dysregulation (APLAID) (two patients) (Table 2).

**Table 2:** Clinical manifestations, molecular profile, treatment, and outcomes of patients with defect affecting the inflammasome (*n* = 21).

**Center**	**Patient (Age of diagnosis (years)/sex)**	**Age of onset of symptoms (months)**	**Clinical features**	**Laboratory features**	**Family history (Consanguinity/Family history)**	**Initial diagnosis**	**Molecular details**	**Treatment details**	**Follow-up duration and outcomes**
**Hyper IgD Syndrome/Mevalonate Kinase Deficiency (MVK) (*****n=*** **8)**									
PGIMER	Pt. 13 (1.33 y/M)	2 months	• Fever • Jaundice • Cholestatic • hepatosplenomegaly anemia • Failure to thrive	CRP: 290 mg/L ESR: 110 mm/hr	Yes (Younger brother of patient 14)	Neonatal cholestasis with sepsis	*MVK* exon 9; c.803 T>C; p.Ile268Thr exon 10; c.976G>A; p.Gly326Arg Missense (phase unknown)	Thalidomide	Alive intermittent episodes of fever present
	Pt. 14 (4.5 y/M)	2 months	• Fever • Jaundice • Anemia,generalized lymphodenopathy, hepatosplenomegaly • Failure to thrive	CRP: 56 mg/L ESR: 38 mm/hr	B/o Pt 13	Sepsis	*MVK* exon 9; c.803 T>C; p.Ile268Thr exon 10; c.976G>A; p.Gly326Arg Missense Same as Pt. 13	Thalidomide	Alive and well
	Pt. 15 (3.5 y/F)	6 months	• Polyarthritis (wrist, elbows, knee), • abdominal pain, Diarrhea, colitis • Anemia, hepatosplenomegaly, generalized lymphadenopathy • Global developmental delay	CRP:160 mg/L ESR:109 mm/h Bone marrow biopsy: Dyserythropoiesis with lymphoid aggregates Gut biopsy: acute on chronic inflammation IgG: 2,079 mg/dL IgA: 303 mg/dL	3rd degree consanguinity, no similar illness in family	JIA/Blau/IBD arthritis	*MVK* exon 6; c.546G>T; p. Leu182Phe Homozygous, Missense	CS, MTX, AZA	Alive and well
SGPGI	Pt. 16 (15 y/M) Ref (14)AM	3 months	• Fever • Rash • Arthralgia • Pleuritis • Peritonitis • Hepatosplenomegaly, generalized lymphadenopathy	CRP: 80 mg/L ESR: 90 mm/hr IgG: 1,465 mg/dL IgA: 1,166 mg/dL IgM: 58.6 mg/dL IgD: 938 mg/dL	Sibling of Pt 17	AID ? HIGD syndrome	*MVK* Exon 11 c.1129G>A p.V3771	NSAIDs Change in treatment: DMARDs stopped	NA
	Pt. 17 (11y /M) (14)AM	2 months	• Fever • Rash • Arthralgia • Hepatosplenomegaly, generalized lymphadenopathy • Peritonitis, adhesions on laparotomy	IgG:1377mg/dL IgA:633mg/dL IgM:119.1mg/dL IgD: 1363mg/dL	Sibling of Pt 16	AID ? HIGD syndrome	*MVK* Exon 11 c.1129G>A p.V3771	NSAIDs Change in treatment: DMARDs stopped	NA
BJWHC	Pt. 18 (3 y/M)	12 months	Fever Petechial rash Recurrent cervical adenitis Sinusitis Hepatosplenomegaly	-	-	-	*MVK* exon 2; c.10G>T; p.Glu4Ter (this is novel) exon 11; c.1129G>A; p.Val377lle Het/AR (this is already known as common Dutch founder variant)	NA	Doing well
	Pt. 19 (0.91 y/M)	15 days	• Fever • Rash Failure to thrive • Dactylitis • Perianal abscess • Otomyocosis	IgG: 2,400 mg/dL IgA: 159 mg/dL IgM: 341 mg/dL CD3: 3,724 CD19: 1,375 CD56: 516 NBT: Normal (98%)	No	PID	*MVK* Exon11; c.1097A>G; Asp366Gly Novel and homozygous Not published	CS	NA
CMC Vellore	Pt. 20 (1y/F)	NA	Recurrent infections Fever Anemia Failure to thrive	IgG: 520mg/dL IgA: 43mg/dL IgM: 39mg/dL TG and Ferritin: increased Fibrinogen: normal Coombs: 1+ NBT: normal	NA	NA	*MVK* Exon7; c.644G>A; p.Arg215Gln Homozygous	NA	NA
**Cryopyrin-Associated Periodic Syndromes (CAPS)/ Muckle –Wells Syndrome (MWS)/Neonatal-Onset Mutisystem Inflammatory Disease (NOMID) (*****n=*** **7)**									
PGIMER	Pt. 21 (10 y /F) (31)AM	1 month	• Recurrent urticarial rash • Arthritis (ankle and wrist) • hypertension, • conjunctivitis, opticatrophy, • nephrotic range anasarca, proteinuria, • hypothyroidism • CSVT	CRP: 19.5 mg/L ESR: 51 mm/hr Renal biopsy: AA Amyloidosis IgG: 623 mg/dL; IgA: 253mg/dL IgM: 282 mg/dL	No	Atypical nephrotic syndrome	*NLRP3* (exon 3; c.1055C > T; p.Ala352Val) Substitution	CS, thalidomide, enalpril, amlodipine	Died due to amyloid associated renal failure
	Pt. 22 (13 y//F)	Infancy	• Fever • Rash • Arthritis with bony overgrowth • Headache, • Short Stature	CRP: 60 mg/L ESR: 98 mm/hr FNAC, abdominal fat pad, amyloidosis	-	Systemic JIA	*NLRP3* exon 3; c.913G>C; p.Asp305His	CS, thalidomide	Alive
	Pt. 23 (11 y/M)	NA	• Fever • Arthritis • Amyloidosis, • renal failure	CRP: 58 mg/L ESR: 89 mm/hr FNAC, abdominal fat pad, renal biopsy, amyloidosis	-	Systemic JIA	*NLRP3* exon 3; c.1792C>T; p.Thr349Ile	CS	Died due to amyloid associated renal failure
	Pt. 24 (6.5 y/M) (32)AM	18 months	• Fever • Erythematous macular non itchy rash, later painful nodular • Seizures with meningitis, • SNHL	CRP: 65 mg/L ESR: 96 mm/hr Skin panniculitis, non specific perivascular dermatitis MR brain: Bilateral Bilateral cerebellar atrophy with mild hydrocephalus	No	Tubercular meningitis	*NLRP3* genetic screening negative for all exons	CS, thalidomide	Died
SGPGI	Pt. 25 (4 y/F)	Since birth	• Fever • Arthritis • Urticaria • Knee flexion contractures • Short stature • Hepatosplenomegaly	CRP:11.7 mg/L ESR: 30 mm/hr	No	Oligo JIA, NOMID	Mutation screening under process	Colchicine	Doing well
	Pt. 26 (5 y/M)	Since birth	• Fever • Arthritis • Urticaria, • Lymphadenopathy, hepatosplenomegaly	CRP:12 mg/L ESR: 90 mm/h IgG:1,590 mg/dL IgA: 275 mg/dL IgM: 109 mg/dL IgE: 409.8 mg/dL	No	NOMID	Mutation screening under process	Colchicine	Doing well
BJWHC	Pt.27 (1.33 y /M)	D1 of life	• Fever • Urticarial rash • Hepatosplenomegaly • Hypertelorism • Macrocephaly • Delay in cognitive milestones	CRP: 10 mg/L ESR: 140.5 mm/h MRI brain: Mild cerebral atrophy with dilated lateral ventricles and cisterns IgG: 1,472 mg/dL IgA: 124 mg/dL IgM: 181 mg/dL IgE: 785 mg/dL	No	AID	*NLRP3* exon 4; c.2263G>A/G>C; p.Gly755Arg	CS, NSAIDs	Died
**NLR Family Pyrin Domain containing 12 (NLRP12) (*****n=*** **2)**									
SGPGI (17)2)	Pt. 28 (4 y/F)	Since birth	• Fever • Diarrhea • Pneumonia • Arthritis	CRP: 54 mg/L ESR: 34 mm/hr	No	PID	*NLRP12* exon 9; c.2935A>G; p.Ser979Gly published	CS	NA
			• Cervical lymphadenopathy and hepatosplenomegaly, • Skin pustules,subcutaneous abscess • Meningitis, SNHL	Gut biopsy: cryptitis with occasional crypt distortion NBT: Normal CD3, CD19, CD56: Normal					
	Pt. 29 (1 y/M)	1 month	• Fever • Urticarial rash, bullous eruptions over fingers, pustular skin lesion • Cervical and axillary lymphadenopathy	CRP: 72 mg/L ESR: 103 mm/hr USG: synovial thickening of joint and both radiocarpal joints. C3/C4: 1.73 mg/dL /0.25 mg/dL	-	AID	*NLRP12* exon3; c.779C>T; p.Thr260Met Heterozygous VUS Not published	CS	Well
**Familial Mediterranean Fever (FMF) (*****n=*** **2)**									
BJWHC	Pt. 30 (0.91y/F)	4 months	Fever Irritability Maculopapular rash Recurrent abdominal pain Hepatomegaly	CRP: 39 mg/L ESR: 53 mm/hr CD3: 4,084 CD19: 2,106 CD56: 128 NBT: 97%	No	AID	*PLCG2* exon 2;c.62C>T; p.Ala21Val het/AD/VUS het/AD/VUS *MEFV* exon 2; c.464G>C; p.Arg155Thr) Het/AD/VUS Not previously published. This is a non-confirmatory variant as per new Eurofever/ PRINTO classification criteria	Colchicine	Doing well
PGIMER	Pt. 31 (1.66 y/M)	9 months	Oral ulcers	CRP: 1.87 mg/L ESR: 37 mm/hr TH17/STAT3: reduced IgE: Normal NBT: Normal CD3, CD19, CD56,CD4, CD8: Normal	N/Yes (oral ulcers in father; Not Screened)	PID (TH17/PSTAT1 defects)	*MEFV* exon 10; c.2177T>C; p.Val726Ala heterozygous, missense. This is a non-confirmatory variant as per new Eurofever/ PRINTO classification criteria	Fluconazole Colchicine	Alive
**PLCG2 associated antibody deficiency and immune dysregulation (APLAID) (*****n=*** **2)**									
PGIMER	Pt. 32 (9 y/F)	24 months	• Generalized Erythematous macular rash, bilateral knee and elbow arthritis, two episodes of intussception, otitis media • Pneumonia, • Recurrent vaginal bleeding • Short stature	HRCT: bilateral hyper inflated lung with fibrotic changes Skin Biopsy: non-specific perivasculitis with no immune deposits ANA: 2+ speckled and nucleolar C3/C4: 73 mg/dL/ <8mg/dL CH50: 166% (69–129) IgG: 1,683 mg/dL(540–1,610) IgA: >594mg/dL (50–240) IgM: 117 mg/dL(50–180) IgE: >10,000 IU/mL CD3: 51.8 (55–78) CD19: 40.89 (10–31) CD56: 3.11	Younger male sibling expired at 9 months, diarrhea, necrotic skin rash	SLE	*PLCG2* exon 22; c.2393 A>G; pAsn798Ser heterozygous, missense	CS, thalidomide, AZA	Died
	Pt. 33 (3 y/M)	4 months	• Fever • Rash (multiple supportive lesions, erythematous plaques pustular lesions, alopecia) along with scars • Photosensitivity • Flexural contractures at small joints of hand, claw hand • Bilateral corneal epithelial defect with corneal ulcers and corneal opacity • Phimosis	CRP: 70 mg/L ESR: 100 mm/hr Skin Biopsy: Epidermis shows Epidermis shows hyperkeratosis, focal neutrophilic crust over stratum corneum, basal cell vacuolation, perivascular infiltrates ANA: Negative IgA: >595 mg/dL IgM: 89 mg/dL IgE: 8,856 mg/dL CD3/CD19/CD56: normal NBT /TH17/STAT3: normal	No	Kindler syndrome, hyper IgE syndrome	*PLCG2* exon 22; c.2393 A>G; pAsn798Ser heterozygous, missense	CS, MTX, IVIg	Died

*AID, Autoinflammatory disorder; ANA, Antinuclear antibodies; AZR, Azathioprine; BD, Behcet disease; BJWHC, Bai Jerbai Wadia Hospital for Children, Mumbai, India; CMC, Christian Medical College and Hospital, Vellore, India; CRP, C-reactive protein; CS, Corticosteroids; CSVT, Cerebral sinovenous thrombosis; CT, Computed tomography; CTA, Computed tomography angiography; CYC, intravenous pulse cyclophosphamide; ESR, Erythrocyte sedimentation rate; FNAC, Fine needle aspiration cytology; HCQS, Hydroxychloroquine; Ig, Immunoglobulin; JIA, Juvenile idiopathic arthritis; MRI, Magnetic resonance imaging; MTX, Methotrexate; MMF, Myocphenolate mofetil; MVL, Mevalonate kinase; NBT, Nitroblue tetrazolium; NLRP3, NLR family pyrin domain containing 3; NLRP12, NLR family pyrin domain containing 12; PID, Primary immunodeficiency disorder; PGIMER, Post Graduate Institute of Medical Education and Research (PGIMER), Chandigarh, India; PLCG2, Phospholipase C gamma 2; Ref, Reference of previously reported paper; SGPGI, Sanjay Gandhi Post Graduate Institute of Medical Sciences (SGPGI), Lucknow, India; SNHL, Sensory neural hearing loss; TG, Triglycerides; Y, years*.

### Hyper IgD Syndrome

Patients with Hyper Ig D syndrome (Patient no. 13–20) had onset of symptoms during infancy (15 days−1 year) with predominant clinical features being fever (7/8), rash (4/8), lymphadenopathy (4/8), hepatosplenomegaly (5/8), and anemia (4/8). Initial diagnosis of neonatal sepsis was considered in two patients (patient no. 13–14). Three patients were found to have the V377I Dutch founder variant and 2 had c.1129G>A variant which is fairly common in South India particularly Kerala (15).

### Cryopyrin-Associated Periodic Syndromes CAPS

**Figure 4:**
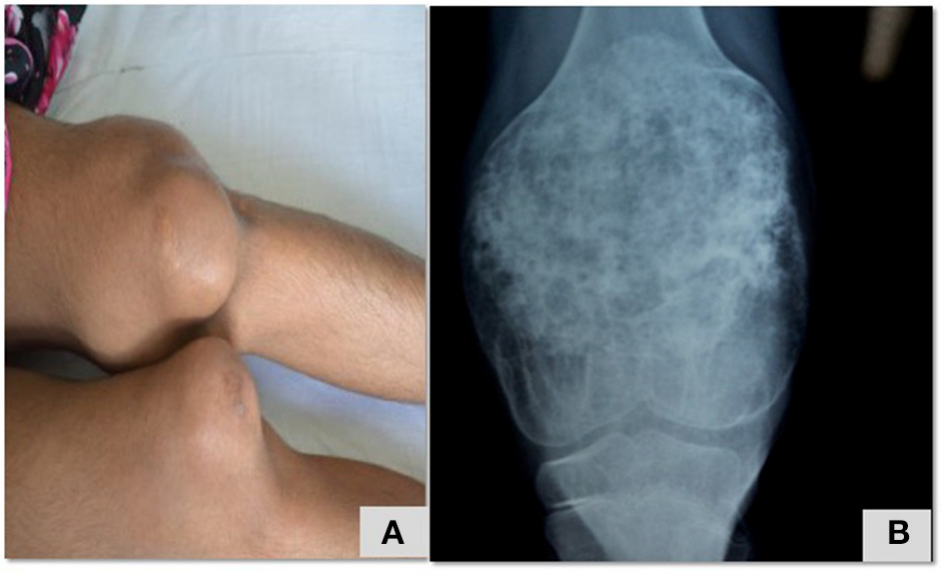
Swelling of knee joint with enlarged, deformed femora and patellae due to overgrowth arthropathy **(A)**, heterogeneously calcified tumor-like protrusions originating from the growth plate **(B)** in a child with NOMID (Table 2; patient 22).

In this cohort we report seven patients with CAPS caused by mutations in NLR family pyrin domain containing 3 (*NLRP3)* gene. All patients had been symptomatic since early infancy but there were significant delays in diagnosis. Age at diagnosis ranged from 15 months to 13 years. Most of these patients were initially diagnosed as JIA. Seven patients had classical phenotype of NOMID with infantile onset of fevers, urticarial rash, arthritis, and progressive deformities with bony overgrowths (Figure 4). Sensory neural hearing loss and headache was found in only1 patient. Of the seven patients, pathogenic variants in *NLRP3* gene were identified in four patients (patient no. 21–23, 27) while no mutation could be identified in patient no 24 on exome sequencing. Molecular studies of two patients (patient 25, 26) are awaited. Three patients (patient no 21,22, and 23) had developed amyloidosis when by the time diagnosis of CAPS was established and two. patients (patient no 21 and 23) succumbed to their illness. Drugs used for treatment included corticosteroids, thalidomide and colchicine as anti-interleukin 1 (anti-IL1) therapy was not easily accessible. 4/7 patients with CAPS died while 3 were alive at the time of this report. Patient no. 22 has been on thalidomide for 12 years which has resulted in normalization of inflammatory parameters but she continues to have significant growth retardation, deformities, and intermittent headaches.

### NLRP12

Variants in NLRP12 gene were identified t in two patients (patient no 28, 29). Both children were symptomatic since early infancy. Patient no. 28 had presented with recurrent episodes of fever, and infections (skin and subcutaneous abscess, diarrhea, meningitis, pneumonia), arthritis, sensorineural hearing loss and hepatosplenomegaly while patient no. 29 in addition had urticarial rash, pustular skin lesions, and lymphadenopathy. Heterozygous mutation in exon 3 in NLR family pyrin domain containing12 **(***NLRP12)* gene was identified. Corticosteroids were used for treatment in patient 28 and is currently well.

### FMF

Clinical profile of patients (Patient no 30, 31) with variants in *MEFV* gene is summarized in Table 2. Patient no. 30 had presented with periodic fever, rash, and abdominal pain. Targeted panel revealed variants of unknown significance in *MEFV* gene and Phospholipase C Gamma 2 (*PLCG 2)* gene. The patient is doing well on colchicine.

Nine months old boy (patient 31) had presented with recurrent oral ulcers. In view of family history of oral ulceration exome sequencing was performed. Heerozygyous issensense mutation in MEFV gene was identified. Symptomatic improvement has been noted after initiation of colchicine.

### APLAID

**Figure 5:**
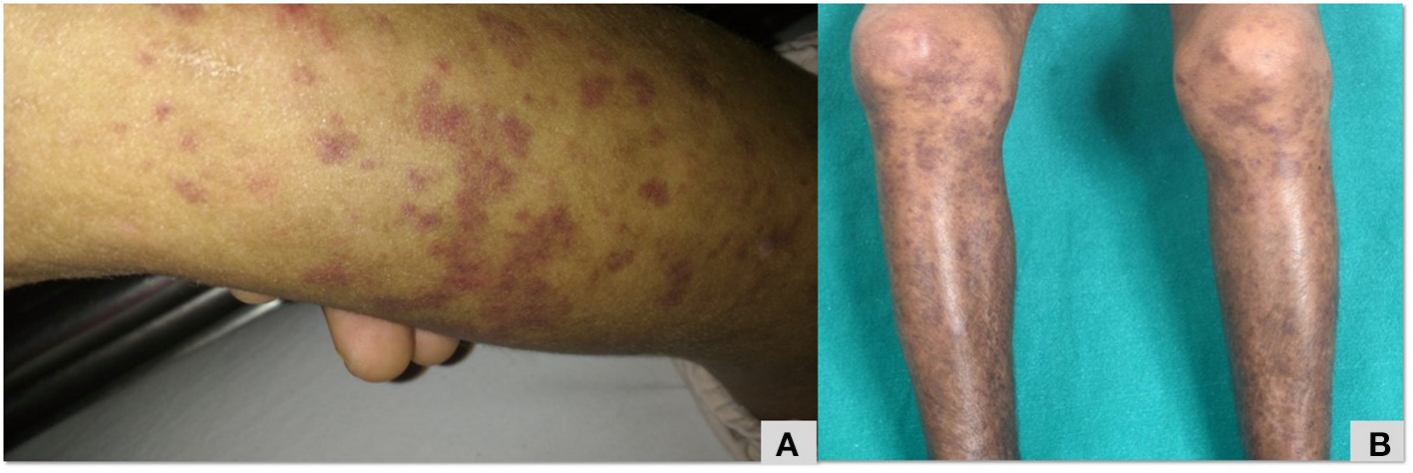
Maculopapular erythematous rash over lower limb **(A,B)** that was initially diagnosed as Henoch Scholein purpura in a patient with PLCG2 (Table 2; patient no 32).

Clinical profiles of patients no. 32, 33 with *PLCG2* variants are summarized in Table 2. Patient no. 32 had erythematous macular rash (Figure 5), large joint arthritis, episodes of intussusception along with recurrent sinopulmonary infections. A *de-novo* heterozygous missense mutation in exon 22 of *PLCG2* gene that resulted in substitution of serine by asparagine at codon 798 (pAsn798Ser), was validated using Sanger sequencing. The Asn798Ser variant has a minor allele frequency of 0.08, 0.07, and 0.16% in the 1,000 genomes, ExAC and internal databases, respectively. The *in-silico* predictions of the variant were found damaging by PolyPhen-2 (HumDiv), damaging by Sorting Intolerant from Tolerant (SIFT), likelihood ratio test (LRT) and Mutation Taster 2.

Patients no. 33 had scaring photosensitive rash and a provisional diagnosis of Kindler syndrome was made (Mahajan et al. manuscript in submission). He was also detected to have same mutations in *PLCG2* gene as patient no. 31. Both these patients were unrelated and belonged to different ethnic backgrounds. They had multiple relapses and both succumbed to their illness.

## Clinical Profile of Patients With Non-Inflammasome Related Conditions

Thirty patients (38%) in our cohort were grouped under non-inflammasome related conditions that included TNF receptor-associated periodic syndrome (TRAPS) (three patients); Deficiency of the Interleukin 1 Receptor Antagonist(DIRA) (two patients); Pyogenic sterile Arthritis, Pyoderma Gangrenosum, Acne syndrome (PAPA) (one patient); A20 haploinsufficiency (four patient); CCA-adding transfer RNA nucleotidyl transferase (TRNT1) (two patients); Caspase recruitment domain-containing protein 14 (CARD 14) (one patients); and Laccase Domain Containing 1(LACC1) (three patients from one family) (Table 3). Patients with Blau syndrome (14 patients are not being presented in this paper (Suri et al., manuscript in submission). Patient with LACC1 has also been reported previously (27) (Table 3).

**Table 3:** Clinical manifestations, molecular profile, treatment and outcomes of patients with non-inflammasome-related conditions (*n* = 16).

**Center**	**Patient (age of diagnosis, years/sex)**	**Age of onset of symptoms**	**Clinical features**	**Laboratory features**	**Family history**	**Initial diagnosis**	**Molecular details**	**Treatment details**	**Follow-up duration and outcomes**
**Tumor necrosis factor receptor-associated periodic syndrome (TRAPS) (*****n=*** **3)**									
PGIMER	Pt. 34 (2.75 y/F)	1 y 9 months	• Periodic fever • Subcutaneous swellings, rash, periorbital edema • Recurrent episodes of abdominal pain	CRP: 41 mg/L ESR: 120 mm/hr IgG: 1,301 mg/dL (270–1,580) IgA: 135 mg/dL (30–130) IgM: 250 mg/dL (50–220) NBT: Normal	Father affected; migratory lymphedema (same mutation)	Periodic fever	*TNFRSF1A* exon3; c.215G>A p.Cys72Tyr previously unreported	CS, NSAID Change in treatment: etanercept	Alive
	Pt. 35 (45 Y/F)	Since adoloscence	• Fever, • Arthralgia • Conjunctivitis, • Pustular psoriasis (recurrent sterile pustular lesions)	CRP: 87 mg/L ESR: 65 mm/hr Skin biopsy: neutrophilic infiltrate in upper spinous and subcorneal layers	No	Pustular psoriasis	*TNFRSF1A* exon9; c.902C>A p.Pro301His Missense Reported in gnomAD. Predicted to be pathogenic by polyphen and SIFT	CS, cyclosporine MTX	Died
Aster CMI	Pt. 36 (10 y/M)	3 months	• Recurrent fevers since early infancy (each episode for 3-4 weeks, afebrile intervals up to 10 days), • Rash over trunk and limbs, • Limb pains and limp, • Abdominal pain • Vomiting • Eye puffiness	CRP: 150 mg/L ESR: 120 mm/hr ANA: Negative	No	TRAPS	*TNFRSF1A* exon 9; c.146A>G; p.Tyr49Cys Previously reported	CS, antimicrobials Change in treatment: Etanercept - partial response Tocilizumab – responded	Alive and doing well
**Deficiency of the interleukin-1 receptor antagonist (DIRA) (*****n=*** **2)**									
PGIMER	Pt. 37 (5 months/F) (26)	21 days	• Reduced movement and pain of left hip, left shoulder, right wrist, bilateral elbows since early infancy (multifocal osteitis) • Pustules	CRP: 110.7 mg/L ESR: 113 mm/h Bone scan: increased uptake in multiple joints (bilateral hip, shoulders, and sternoclavicular joints, lower ribs near costochondral junction and left elbow) X-ray: osteolytic lesions at humerus, left proximal femur, ribs and clavicle Bone biopsy: Bone inflammation	No	DIRA	*IL1RN* deletion, at chr2_hg19_113,865,011 and chr2_hg19_113,887,227 homozygous 22,216bp deletion spans the first four exons of IL1RN, Parents carrier for same mutation (NM_173843) Homozygous deletion Exon 1-4 deletion	Change in treatment done: Anakinra	Well
	Pt. 38 (2.58 y/M)	7 days	Paucity of bilateral upper limb movements since day 7 of life Pustular lesions	CRP: 1.8 mg/L ESR: 8 mm/hr X-ray: bilateral humerus, clavicle and rib metaphyseal widening,	No	DIRA	Mutation for ILRN deletion as in patient 36 screened but not found	CS	Alive, healed lesions
**Pyogenic Arthritis, Pyoderma gangrenosum and Acne (PAPA) (*****n=*** **1)**									
PGIMER	Pt. 39 (5 y/F)	2.5 y	• Fever • Pyoderma gangrensosum • Colitis • Multiple abscess • Pus drainage, fistula, oral ulcers, pustules • Abdominal pain • Recurrent diarrhea	CRP: 101 mg/L ESR: 35 mm/hr Platelets: 964 × 109/L Colonoscopy: ileocecal valve thickened and distorted. Ileum shows active ulceration, cobble stone appearance, pseudo-polyp. Alteration of vascular pattern in cecum and ascending colon. Few active ulcers in hepatic flexure, transverse colon, recto sigmoid junction with pseudo-polyps Impression: Crohn's disease or tuberculous colitis Gut biopsy: Crohn's disease ANA, ANCA: negative C3/C4: 182/23 IgG: 869 NBT, CD3: Normal	-	Crohn's disease	*PSTPIP1* exon3; c.203C>T; p.Thr68Met Missense Place: Gasilini, italy	ATT, CS, infliximab, AZA	Died
**A20 haploinsufficiency (TNFAIP3) (*****n=*** **4)**									
PGIMER	Pt. 40 (6 y/F)	6 M	• Recurrent fever • Oro-genital ulcers • Ocular inflammation, blurring of vision • Headache • Papilledema • Abdominal pain • Arthritis • Colitis	CRP: 73.9 mg/L ESR: 26 mm/hr MR brain: type 2 Arnold Chiari malformation, HLAB51: positive ANA, ANCA: negative Gut Biopsy: no vasculitis	Younger brother has recurrent oral ulcers since 8 months age; Mother heterozygous for same variant	Behcet disease	*TNFAIP3* exon 7;c.1504C>T; p.Arg502Trp Heterozygous missense	colchicine, AZA	Alive and well
CMC Vellore	Pt. 41 (7 y/M)	NA	• Autoinflammatory syndrome • Inflammatory ulcers duodenum to caecum, gastritis	IgG: NA IgA: 579 mg/dL IgM: NA IgE: NA CD3:487 CD19: 22 CD56: 410	NA	NA	*TNFAIP3* exon7; c.1316_1317del; p.Gly440ArgfsTer4 Heterozygous Novel Likely pathogenic	NA	NA
CMC Vellore	Pt. 42 (7y/M)	NA	• AIHA, • Skin rashes • Immune deficiency	IgG: 2148mg/dL IgA: 145mg/dL IgM: 14mg/dL IgE: NA Direct coombs test 3+, Ferritin normal. No increase in Double negative TCRαβ+ T cells	NA	NA	*TNFAIP3* exon8; c.2036T>C; p.Ile679Thr Heterozygous VUS	NA	NA
CMC Vellore	Pt. 43 (3 y/M)	NA	Osteomyelitis/CGD	NA	NA	NA	• *TNFAIP3* • exon7; c.1807G>A; • p.Gly603Arg • Heterozygous • VUS	NA	NA
**TRNT1 deficiency (Sideroblastic anemia, immune deficiency, periodic fever, delay) (SIFD) (*****n=*** **2)**									
ASTER CMI	Pt. 44 (3y/M)	6 months	• Recurrent fever (each episode for 4-7 days and recur twice a month) • Diarrhea • Vomiting • Panhypogmmaglobulinmeia	IgG: 223 mg/dL IgA: 17mg/dL IgM: 24 mg/dL CD3: 77% (1927) CD19: 2.5% (62) CD56: 18% (460)	Two brothers had died within the first 2 years of life with recurrent fever	X-linked agammaglobulinemia	*TRNT1* exon 2; c.143_144insTT p.Thr49Ter and exon 7;c.1043A>T p.Asp348Val compound heterozygous mutation	Replacement IVIg	Doing well
CMC Vellore	Pt. 45 (5y/M)	NA	• Hypogammaglobulinemia • Bronchiectasis	IgG: 478 mg/dL IgA: 31 mg/dL IgM: 50mg/dL IgE: 22.8 mg/dL CD3: 2,897 CD19: 96 CD56: 747 Elevated ferritin	NA	NA	*TRNT1* exon5; c.569G>T; p.Arg190Ile Homozygous	NA	NA
**CARD14 mediated psoriasis (CAMPS) (*****n=*** **1)**									
CMC Vellore	Pt. 46 (8 y/M)	NA	Psoriasis	NA	NA	NA	*CARD14* exon7; c.458G>C; p.Cys153Ser homozygous	NA	NA
**Laccase Domain Containing 1 (LACC1) defect (*****n=*** **3)**									
PGIMER	Pt. 47 (5.75y/F) (27)	9 M	• Polyarticular joint disease. • Joint symptoms with involvement of knee and ankle and rapidly progressed to involve small joints and cervical spine, multiple joint involvement, pain, deformities and contractures, bed bound, stunted, nail dystrophy, marked swelling, deformity of large and small joints	X-ray: osteopenia, erosion of vertebrae without any platyspondyly RA Factor: positive	Sibling of Pt. 49 and 50	Torg Winchester syndrome, Pseudorheumatoid chondrodysplasia and Familial inflammatory arthropathy	*LACC1* exon4; c. 832G>C, p.Ala278Pro Parents heterozygous for the same	Naproxen, CS, MTX	Doing satisfactory
	Pt. 48 (3y/F) (27)	9 M	• Polyarticular joint disease. • Joint symptoms with involvement of knee and ankle and rapidly progressed to involve small joints and cervical spine, multiple joint involvement, pain, deformities and contractures, bed bound, stunted, nail dystrophy, marked swelling, deformity of large and small joints	X-ray: osteopenia, erosion of vertebrae without any platyspondyly	Sibling of Pt. 48 and 50	Similar to Pt 48	Same as Pt 48	Naproxen, CS, MTX	Doing satisfactory
	Pt. 49 (0.91y/F) (27)	9 M	• Polyarticular joint disease. • Joint symptoms with involvement of knee and ankle and rapidly progressed to involve small joints and cervical spine, multiple joint involvement, pain, deformities and contractures, bed bound, stunted, nail dystrophy, marked swelling, deformity of large and small joints	X-ray: ostopenia, erosion of vertebrae without any platyspondyly	Sibling of Pt. 48 and 49	Similar to Pt 48	Same as Pt 48	Naproxen, CS, MTX	Doing satisfactory

*ANA, Antinuclear antibodies; Aster CMI, Aster CMI Hospital, Bengaluru, India; AZR, Azathioprine; CARD14, Caspase recruitment domain family member 14; CMC, Christian Medical College and Hospital, Vellore, India; CRP, C-reactive protein; COPA, Coatamer complex 1 protein alpha subunit; CS, Corticosteroids; CT, Computed tomography; CTA, Computed tomography angiography; ESR, Erythrocyte sedimentation rate; HCQS, Hydroxychloroquine; IL1RN, Interleukin 1 Receptor Antagonist; IVIg, Intravenous immunoglobulin; JIA, Juvenile idiopathic arthritis; LACC1, Laccase domain containing 1; MRI, Magnetic resonance imaging; MTX, Methotrexate; PGIMER, Post Graduate Institute of Medical Education and Research (PGIMER), Chandigarh, India; PSTPIP1, Proline-serine-threonine phosphatase interacting protein 1; Reference of previously reported paper; TNFAIP3, TNF alpha induced protein 3; TNFRSF1A, TNF receptor superfamily member 1 A; TRNT1, TRNA nucleotidyl tranferase 1; Y, year*.

### TRAPS

**Figure 6:**
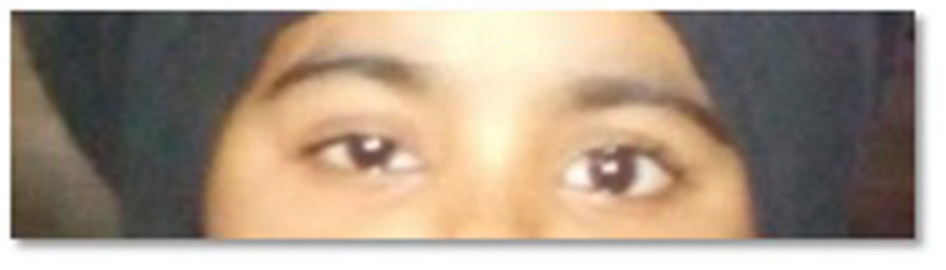
Unilateral periorbitaledema without conjunctivitis noted in child with TRAPS during flares (Table 3; Patient no 34).

Patient no. 34 had recurrent episodes of fever lasting for 2–3 weeks every 3–4 months with rash since 18 months of age. These f episodes were associated with pain abdomen, myalgias, arthritis, periorbital edema (Figure 6), and subcutaneous swellings. She had received multiple courses of antimicrobials in view of marked polymorphonuclear leukocytosis. Her father was also symptomatic and used to have fever and intermittent subcutaneous swelling and rash. Father was diagnosed to have acute rheumatic fever in childhood. In view of periodic fever, with systemic manifestations and family history suggestive of autosomal dominant disorder, diagnosis of TRAPS was proffered and confirmed on exome sequencing. She was initially managed with corticosteroids followed by injection etanercept. She remains well at follow up.

A 45 years old female (patient no. 35) who had been under follow-up of Dermatology services was diagnosed to have pustular psoriasis since early adolescence. She would have intermittent flares with rash, fever and arthritis. I Her disease was refractory to methotrexate, cyclosporin and corticosteroids. In view of recurrent episodes of fevers, markedly elevated inflammatory parameters, sterile neutrophilic infiltrates on skin biopsy, a possibility of autoinflammatory disease was considered. Whole exome sequencing revealed variant in TNF Receptor Superfamily Member 1A(*TNFRSF*1A) mutation. She could not be initiated on biological agents due to financial constraints and succumbed to her illness 2 years after diagnosis was established.

A 10-year-old boy (patient no 36) had presented with high grade fever without focus. He reported having febrile episodes lasting for 3–4 weeks with variable afebrile periods since early childhood. These episodes were associated with rash over the trunk and limbs, myalgia and limp, abdominal pain, vomiting, and periorbital swelling. The inflammatory parameters were elevated and targeted panel for autoinflammatory diseases confirmed the diagnosis of TRAPS. The patient demonstrated partial response to etanercept which was changed to tocilizumab to which he responded well.

### DIRA

Patient no 37 as has been previously reported (26), was the first Indian patient with large deletion in Interleukin 1 Receptor Antagonist (*IL1RN*) gene. She is doing well on Anakinra at 6 years of follow-up supported by National Institutes of Health, USA.

Patient no 38 had presented at day 7 of life with paucity of movement of both upper limbs. Inflammatory parameters were increased with sterile blood cultures. X-rays showed bilateral humerus, rib and clavicular involvement. He was treated with oral prednisolone 2 mg/Kg with slow tapper over 4 months. He responded dramatically and bone lesions healed. He developed pustules at follow up. Deletion in IL1RN gene as in patient no 37 was not detected on Western blot analysis. Results of whole exome sequencing are awaited. He is currently doing well and off corticosteroids. However, ESR remains elevated.

### PAPA Syndrome

A 4-years old girl (patient no 39) had been unwell for 2.5 years when she presented with periodic fevers associated with painful oral ulcers, abdominal pain with hematochezia and colitis. Over the years, she developed multiple pyoderma gangrenosum lesion over extremities, angle of mouth and gluteal region that caused complete destruction of left cheek and lower lip. The lesion were difficult to heal and resulted in fistulae formation. She was initially suspected to have inflammatory bowel disease and oral prednisolone and azathioprine were initiated. Injection infliximab (3 doses) were also commenced. There was partial response in skin lesions and colitis initially. However, lesions reoccurred, and she succumbed to her illness.

### A20 Haploinsufficiency

**Figure 7:**
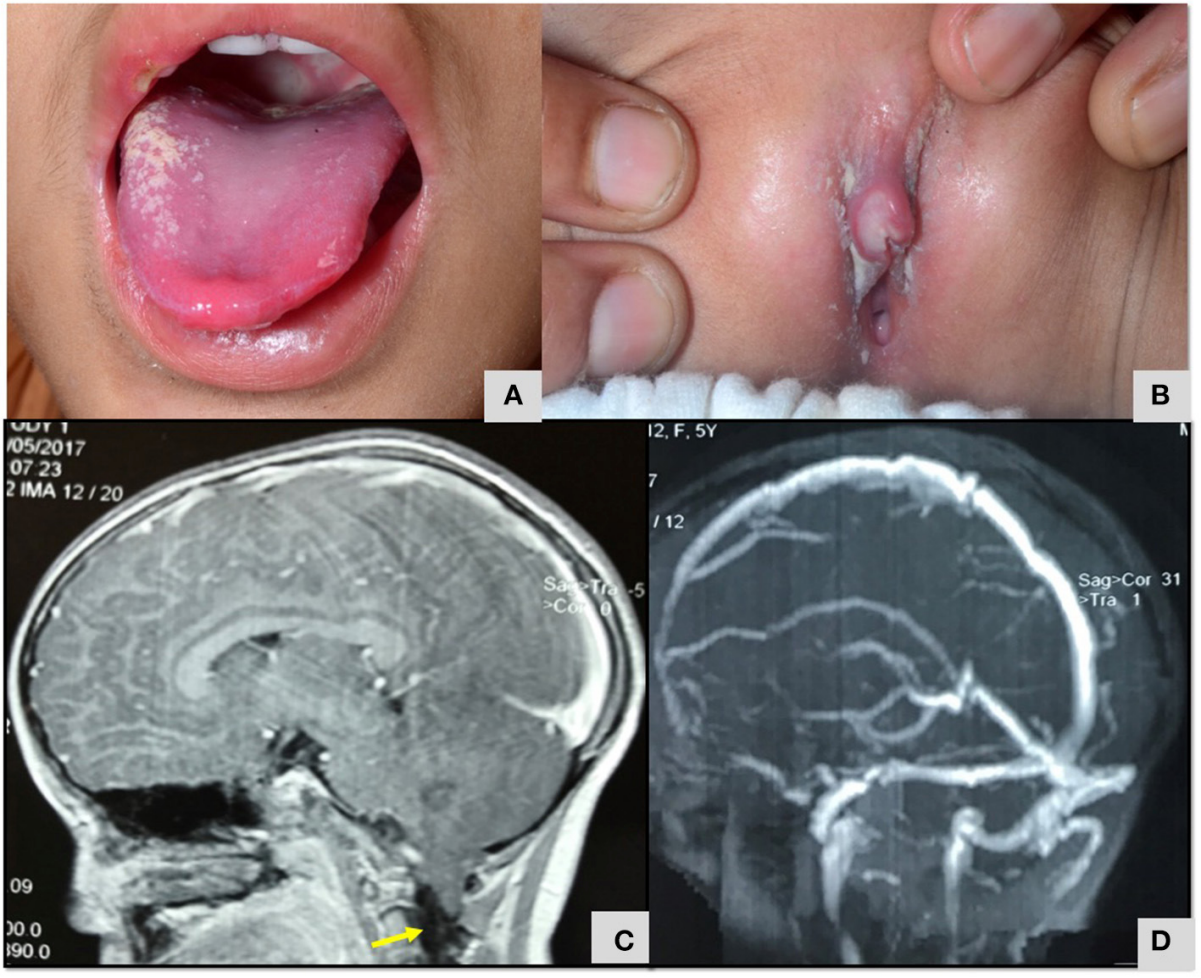
**(A,B)** Oral cavity erythema and ulceration and genital ulcers in child with A20 haploinsufficiency (Table 3; Patient no 40). **(C)** MRI Brain T2 weighted sagittal section images demonstrating type 2 Arnold Chiari malformation (Table 3; Patient no 40). **(D)** MR venography of brain demonstrated normal flow and no evidence of cerebral sinus venous thrombosis (Table 3; Patient no 40).

Patient 40 was 2 years old when she had presented with recurrent oral ulcers and genital ulcers (Figure 7). She had colitis, refectory ulcers requiring repeated hospitalization. Markers of inflammation were elevated, and Human Leucocyte Antigen 51 (HLA B 51) allele was detected. Considering a possibility of Bechet's disease, she was commenced on corticosteroid and azathioprine. At 5 years, she was readmitted with persistent headache, blurring of vision and relapse of oro-genital ulcers. She had papilledema and magnetic resonance imaging (MRI) of brain revealed type 2 Arnold Chiari malformation. When younger brother also developed recurrent oral ulcers, genetic studies were performed in the index patent and targeted panel revealed a novel variant in Tumor necrosis factor alpha-induced protein 3 (*TNFAIP3)* gene (Table 3). She is doing well on follow up. Mother is carrier for the same variant while the younger sibling does not carry this variant. Patient 41 had also presented with early onset inflammatory bowel disease and oral ulcers and a novel variant in *TNFAIP3)* gene was found.

### TRNT1 Deficiency

Patient no. 44, had presented with recurrent fever, and diarrhea. Two elder brothers earlier had died. He was evaluated for primary immune deficiency and was noted to have pan hypogammaglobulinemia. A provisional diagnosis of X-linked agammaglobulinemia was made and intravenous immunoglobulin replacement initiated. Exome sequencing revealed a compound heterozygous mutation in exon 2 of *TRNT1* gene. Similarly, patient no. 48 also had recurrent infections, bronchiectasis and hypogammaglobulinemia.

**CARD 14:** Patient 46had early onset difficult to treat psoriasis and was found to have mutations in CARD 14 gene.

## Discussion

SAID were first recognized in 1999 (4). Over the last two decades, knowledge and recognition of SAID has grown at an unprecedented speed and there have been a plethora of publications on this subject (3, 12, 13, 22). Significant improvement in understanding of genetic and pathogenic mechanisms of SAIDs has resulted in remarkable progress in their management. However, the darta from India is limited (14–18, 26, 27, 33–40). There is no national registry for SAID and there is lack of knowledge on nationwide burden of these diseases. This manuscript is the first attempt to collate data from various centers involved in care of patients with SAID and highlight diagnostic difficulties, treatment, and outcomes of such patients in India.

SAID display wide range of clinical manifestations and can affect almost every organ system. They are uncommon and difficult to diagnose clinically. Overlapping clinical features of these disorders with other relatively common rheumatological disorders often lead to delayin diagnosis. At Chandigarh, we diagnosed our first child with NOMID in 2005. This child was being managed as systemic onset JIA for over 10 years. Similarly, patients with Blau syndrome were initially treated as JIA with uveitis, and patients with DADA2 as PAN. Other initial diagnosis included systemic lupus erythematosus, inflammatory bowel disease and Bechet's disease. With improved awareness amongst internists and pediatrician along with availability of affordable diagnostic techniques, these syndromes are now being suspected and diagnosed early.

Diagnosis of most SAID is based on clinical suspicion, family history and demonstration of elevated inflammatory parameters (ESR, CRP, serum amyloid A protein). Distinct interferon signatures and cytokine patterns may be helpful biomarkers to stratify and monitor patients. However, interpretation and standardization of these tests is difficult. Despite expansion of various laboratory investigations, these investigations are not yet available for clinical use in India. Genetic analysis is needed in all patients for confirmation of diagnosis. In the past, most genetic studies were performed in collaboration with international centers. In the last 5 years, molecular diagnostic techniques were established at PGIMER Chandigarh and National Institute of Immunohematology Mumbai, which are Indian Council of Medical Research (ICMR) recognized Centers for Advanced Research (CAR) in Primary Immune Deficiency Diseases. However, diagnostic facilities are still limited. At PGIMER, among SAID, we can perform Sanger sequencing for *ADA2* and *NOD2* genes. In recent years, NGS based targeted autoinflammatory panels are available in commercial laboratories, albeit expensive. Interpretation of data and functional validation of variants of unknown significance (VUS) detected remains a challenge.

Management of SAID is aimed at suppression of systemic inflammation. Colchicine and glucocorticoids have been traditionally used to treat SAID. However, with improved knowledge and understanding of pathogenic mechanisms of autoinflammation and availability of specific targeted immunotherapies, treatment strategies have been completely revolutionized (41, 42). Anti-IL-1 drugs (anakinra, canakinumab, and rilonacept), have become standard of care for most inflammasomopathies. These agents successfully control inflammation and improve growth and quality of life. Other biologic agents used include anti-TNF drugs (etanercept), anti-IL-6 drugs (tocilizumab), and Janus kinase inhibitors (tofacitinib, baricitinib, and ruxolitinib). Treatment of SAID is extremely challenging in resource constrained settings. Anti-IL-1 drugs are not readily available in India and other developing countries. These drugs have to be imported on a “named-patient-basis” and are exorbitantly expensive. Some biosimilar molecules like anti TNF (adalimumab, infliximab) and anti IL6 (tocilizumab) are available alternative therapies. Though these molecules are cost cheaper in India when compared to Western countries, yet they remain well beyond the scope of average Indian family. Thus, corticosteroids and other conventional immunosuppressive agents still form the main stay of therapy. Patients often require higher doses as the disease progresses and they often develop corticosteroid related side effects. Off late, hematopoietic stem cell transplant (HSCT) is also emerging as a curative option for some SAID (43, 44). However, none of our patients received HSCT.

AID related morbidity and mortality continues to be high. In our cohort (8/49) have died at the time of analysis due to non-availability of treatment and development of complications. Amyloidosis had already developed in four patients at the time of diagnosis and it remains an important cause of death.

There are several limitations of this study. It is a case-based record review report from major primary immunodeficiency diseases centers across the country. Data from various individual rheumatology units could not be collated. Moreover, shared data were not uniform from all centers. Patients with unclassified SAID and patients in whom molecular diagnosis could not be established were excluded from the study.

This is a first comprehensive multicentric report of patients with SAID from India. Varied clinical and molecular spectrum has been reported. Considerable delays in diagnosis were recognized. Application of NGS based targeted panels and whole exome sequencing has helped in identifying known as well as novel gene defects. Establishment of diagnosis in a patient enabled early diagnosis of other family members and provided an opportunity for prenatal diagnosis. Although, ability to diagnose SAID has improved, non-availability of expensive immunotherapies remains a major drawback. In India corticosteroids and conventional immunosuppressive agents continue to remain corner stones for treatment. Lack of availability of targeted immunotherapies for treatment prevents the initiation of effective treatments that can change patients' lives. SAID continue to result in significant morbidity and mortality.

To conclude, more efforts are needed to enhance awareness of autoinflammatory diseases among health care professionals and there is an urgent need to make life saving drugs universally available.

## Data Availability Statement

The original contributions presented in the study are included in the article/supplementary material, further inquiries can be directed to the corresponding author/s.

## Ethics Statement

The studies involving human participants were reviewed and approved by Institute Ethics Committee, PGIMER, Chandigarh (Ref No: INT/IEC/2021/SPL-264). Written informed consent from the participants' legal guardian/next of kin was not required to participate in this study in accordance with the national legislation and the institutional requirements. Written informed consent was obtained from the minor(s)' legal guardian/next of kin for the publication of any potentially identifiable images or data included in this article.

## Disclosure

RG-M and AA are supported by the Intramural Research Program of NIAID, National Institutes of Health, Bethesda, Maryland, USA.

## Author Contributions

DS, AR, AJ, PV, AG, and RP: data collection, writing of initial draft, editing of manuscript at all stages of its production, patient management, and review of literature. DS, AR, AJ, PV, AG, RP, VJ, KA, RK, GA, AA, SP, FN, BG, EE, MD, PT, VG, AP, SB, and SK: data collection, management of patients, and review of final manuscript. AR, VJ, KA, RK, SP, FN, BG, and ES: genetic evaluation and data collection. MG, IC, AAdJ, and RG-M: genetic evaluation, review of final manuscript, and critical revision. AR, SB, SK, MG, IC, AAdJ, RG-M, and SS: genetic evaluation and review of the final manuscript. MH: performed ADA2 levels in patients with DADA two patients. DS, AR, and SS: patient management, review of literature, editing and critical revision of manuscript at all stages of its production, and final approval of manuscript. All authors contributed to the article and approved the submitted version.

## Conflict of Interest

The authors declare that the research was conducted in the absence of any commercial or financial relationships that could be construed as a potential conflict of interest.

## References

[1] Kastner DL Aksentijevich I Goldbach-Mansky R. Autoinflammatory disease reloaded: a clinical perspective. Cell. (2010) 140:784–90. 10.1016/j.cell.2010.03.002 20303869 PMC3541025

[2] Marino A Tirelli F Giani T Cimaz R. Periodic fever syndromes and the autoinflammatory diseases (AIDs). J Transl Autoimmun. (2020) 3:100031. 10.1016/j.jtauto.2019.100031 32743516 PMC7388371

[3] Stoffels M Kastner DL. Old dogs, new tricks: monogenic autoinflammatory disease unleashed. Annu Rev Genomics Hum Genet. (2016) 31:245–72. 10.1146/annurev-genom-090413-025334 27362340

[4] McDermott MF Aksentijevich I Galon J McDermott EM Ogunkolade BW Centola M. Germline mutations in the extracellular domains of the 55 kDa TNF receptor, TNFR1, define a family of dominantly inherited autoinflammatory syndromes. Cell. (1999) 97:133–44. 10.1016/S0092-8674(00)80721-7 10199409

[5] Aksentijevich I Centola M Deng Z Sood R Balow JE Wood G. Ancient missense mutations in a new member of the RoRet gene family are likely to cause familial Mediterranean fever. The international FMF Consortium. Cell. (1997) 90:797–807. 10.1016/s0092-8674(00)80539-5 9288758

[6] Samuels J Aksentijevich I Torosyan Y Centola M Deng Z Sood R. Familial Mediterranean fever at the millennium. Clinical spectrum, ancient mutations, and a survey of 100 American referrals to the National Institutes of Health. Medicine (Baltimore). (1998) 77:268–97. 10.1097/00005792-199807000-00005 9715731

[7] Ben-Chetrit E Gattorno M Gul A Kastner DL Lachmann HJ Touitou I. Consensus proposal for taxonomy and definition of the autoinflammatory diseases (AIDs): a Delphi study. Ann Rheum Dis. (2018) 77:1558–65. 10.1136/annrheumdis-2017-212515 30100561

[8] Gattorno M Hofer M Federici S Vanoni F Bovis F Aksentijevich I. Classification criteria for autoinflammatory recurrent fevers. Ann Rheum Dis. (2019) 78:1025–32. 10.1136/annrheumdis-2019-215048 31018962

[9] Navallas M Inarejos Clemente EJ Iglesias E Rebollo-Polo M Zaki FM Navarro OM. Autoinflammatory diseases in childhood, part 1: monogenic syndromes. Pediatr Radiol. (2020) 50:415–30. 10.1007/s00247-019-04536-9 32065272

[10] Navallas M Inarejos Clemente EJ Iglesias E Rebollo-Polo M Hernández JC Navarro OM. Autoinflammatory diseases in childhood, part 2: polygenic syndromes. Pediatr Radiol. (2020) 50:431–44. 10.1007/s00247-019-04544-9 32065273

[11] Tangye SG Al-Herz W Bousfiha A Chatila T Cunningham-Rundles C Etzioni A. Human inborn errors of immunity: update on the classification from the international union of immunological societies expert committee. J Clin Immunol. (2020) 40:24–64. 10.1007/s10875-019-00737-x 31953710 PMC7082301

[12] Oda H Kastner DL. Genomics, biology, and human illness: advances in the monogenic autoinflammatory diseases. Rheum Dis Clin North Am. (2017) 43:327–45. 10.1016/j.rdc.2017.04.011 28711137 PMC5564228

[13] Stone DL Beck DB Manthiram K Park YH Chae JJ Remmers E. The systemic autoinflammatory diseases: coming of age with the human genome. J Allergy Clin Immunol. (2020) 146:997–1001. 10.1016/j.jaci.2020.09.014 32987090 PMC11008603

[14] Lawrence A Hol F Aggarwal A Drenth JPH. Hyperimmunoglobulinaemia D syndrome in India: report of two siblings with a novel mutation. Ann Rheum Dis. (2006) 65:1674–6. 10.1136/ard.2006.054494 17105862 PMC1798448

[15] Govindaraj GM Jain A Peethambaran G Bhoyar RC Vellarikkal SK Ganapati A. Spectrum of clinical features and genetic variants in mevalonate kinase (MVK) gene of South Indian families suffering from Hyperimmunoglobulin D Syndrome. PLoS ONE. (2020) 15:e0237999. 10.1371/journal.pone.0237999 32822427 PMC7442240

[16] Babu K Rao AP. Clinical profile in genetically proven blau syndrome: a case series from South India. Ocul Immunol Inflamm. (2020). 10.1080/09273948.2020.1746353. [Epub ahead of print]. 32293936

[17] Gupta L Ahmed S Singh B Prakash S Phadke S Aggarwal A. Novel NLRP12 variant presenting with familial cold autoimmunity syndrome phenotype. Ann Rheum Dis. (2019). 10.1136/annrheumdis-2019-216158 [Epub ahead of print]. 31446425

[18] Jindal AK Pilania RK Suri D Gupta A Gattorno M Ceccherini I. A young female with early onset arthritis, uveitis, hepatic, and renal granulomas: a clinical tryst with Blau syndrome over 20 years and case-based review. Rheumatol Int. (2021) 41:173–81. 10.1007/s00296-019-04316-6 31062074

[19] Anjani G Jindal AK Prithvi A Kaur A Rawat A Sharma M. Deforming polyarthritis in a north indian family-clinical expansion of STING-Associated Vasculopathy with Onset in Infancy (SAVI). J Clin Immunol. (2021) 41:209–11. 10.1007/s10875-020-00872-w 32974768

[20] Jindal AK Pilania RK Rawat A Singh S. Primary immunodeficiency disorders in india-a situational review. Front Immunol. (2017) 8:714. 10.3389/fimmu.2017.00714 28674536 PMC5474457

[21] Pilania RK Chaudhary H Jindal AK Rawat A Singh S. Current status and prospects of primary immunodeficiency diseases in Asia. Genes Dis. (2020) 7:3–11. 10.1016/j.gendis.2019.09.004 32181271 PMC7063407

[22] Wekell P Berg S Karlsson A Fasth A. Toward an inclusive, congruent, and precise definition of autoinflammatory diseases. Front Immunol. (2017) 8:497. 10.3389/fimmu.2017.00497 28496446 PMC5406409

[23] Brodszki N Frazer-Abel A Grumach AS Kirschfink M Litzman J Perez E. European Society for Immunodeficiencies (ESID) and european reference network on rare primary immunodeficiency, autoinflammatory and autoimmune diseases (ERN RITA) complement guideline: deficiencies, diagnosis, and management. J Clin Immunol. (2020) 40:576–91. 10.1007/s10875-020-00754-1 32064578 PMC7253377

[24] Lepelley A Martin-Niclós MJ Le Bihan M Marsh JA Uggenti C Rice GI. Mutations in COPA lead to abnormal trafficking of STING to the Golgi and interferon signaling. J Exp Med. (2020) 217:e20200600. 10.1084/jem.20200600 32725128 PMC7596811

[25] Deng Z Chong Z Law CS Mukai K Ho FO Martinu T. A defect in COPI-mediated transport of STING causes immune dysregulation in COPA syndrome. J Exp Med. (2020) 217:20201045. 10.1084/jem.20201045 32725126 PMC7596814

[26] Mendonca LO Malle L Donovan FX Chandrasekharappa SC Montealegre Sanchez GA Garg M. Deficiency of interleukin-1 receptor antagonist (DIRA): report of the first indian patient and a novel deletion affecting IL1RN. J Clin Immunol. (2017) 37:445–51. 10.1007/s10875-017-0399-1 28503715 PMC8420971

[27] Singh A Suri D Vignesh P Anjani G Jacob P Girisha KM. LACC1 gene mutation in three sisters with polyarthritis without systemic features. Ann Rheum Dis. (2020) 79:425–6. 10.1136/annrheumdis-2019-216263 31811059

[28] Ben-Ami T Revel-Vilk S Brooks R Shaag A Hershfield MS Kelly SJ. Extending the clinical phenotype of adenosine deaminase 2 deficiency. J Pediatr. (2016) 177:316–20. 10.1016/j.jpeds.2016.06.058 27514238

[29] Sharma A Naidu G Sharma V Jha S Dhooria A Dhir V. Deficiency of adenosine deaminase 2 in adults and children: experience from India. Arthritis Rheumatol. (2021) 73:276–85. 10.1002/art.41500 32892503 PMC7902299

[30] Banday AZ Kaur A Jindal AK Patra PK Guleria S Rawat A. Splice-site mutation in COPA gene and familial arthritis - a new frontier. Rheumatology. (2021) 60:e7–e9. 10.1093/rheumatology/keaa354 32778887

[31] Pandiarajan V Gupta A Rowczenio D Hawkins P Muralidaran C Tiewsoh K. Nephrotic syndrome as a presenting feature in a child with NLRP3 mutation. J Clin Rheumatol. (2018). 10.1097/RHU.0000000000000942 [Epub ahead of print]. 30431487

[32] Sankhyan N Kalra V Aksentijevich I Kabra M Gulati S Sharma S. Atypical late presentation in neonatal-onset multisystem inflammatory disease (NOMID). J Pediatr Neurol. (2015) 7:301–5. 10.3233/JPN-2009-0297

[33] Janarthanan M Poddar C Sudharshan S Seabra L Crow YJ. Familial Blau syndrome: First molecularly confirmed report from India. Indian J Ophthalmol. (2019) 67:165–7. 10.4103/ijo.IJO_671_18 30574935 PMC6324106

[34] Khubchandani R Aksentijevich I. Deficiency of adenosine deaminase 2 (DADA2) - a new autoinflammatory disease with multisystem features. Indian Pediatr. (2020) 2020:S097475591600231. 10.1007/s13312-020-2041-1 32796151

[35] Correa ARE Gupta N Bagri N Vignesh P Alam S Yamaguchi S. Mevalonate kinase deficiency as cause of periodic fever in two siblings. Indian Pediatr. (2020) 15:180–1. 10.1007/s13312-020-1742-9 32060250

[36] Sandhya P Vellarikkal SK Nair A Ravi R Mathew J Jayarajan R. Egyptian tale from India: application of whole-exome sequencing in diagnosis of atypical familial Mediterranean fever. Int J Rheum Dis. (2017) 20:1770–5. 10.1111/1756-185X.13042 28211254

[37] Nair SB Chavan PP Athalye AS Aksentijevich I Khubchandani RP. Detection of a novel mutation in NLRP3/CIAS1 gene in an Indian child with Neonatal-Onset Multisystem Inflammatory Disease (NOMID). Clin Rheumatol. (2019) 38:403–6. 10.1007/s10067-018-4225-9 30066283

[38] Raghawan AK Ramaswamy R Radha V Swarup G. HSC70 regulates cold-induced caspase-1 hyperactivation by an autoinflammation-causing mutant of cytoplasmic immune receptor NLRC4. Proc Natl Acad Sci USA. (2019) 22;116:21694–703. 10.1073/pnas.1905261116 31597739 PMC6815140

[39] Gupta A Tripathy SK Phulware RH Arava S Bagri NK. Cryopyrin-associated periodic fever syndrome in children: a case-based review. Int J Rheum Dis. (2020) 23:262–70. 10.1111/1756-185X.13772 31858722

[40] Ghosh K Mishra K Shah A Patel P Shetty S. Novel deleterious sequence change in the NLRP12 gene in a child with the autoinflammatory syndrome, joint hypermobility and cutis Laxa from India. Mediterr J Hematol Infect Dis. (2019) 11:e2019018. 10.4084/mjhid.2019.018 30858956 PMC6402545

[41] Soriano A Soriano M Espinosa G Manna R Emmi G Cantarini L. Current therapeutic options for the main monogenic autoinflammatory diseases and PFAPA syndrome: evidence-based approach and proposal of a practical guide. Front Immunol. (2020) 11:865. 10.3389/fimmu.2020.00865 32655539 PMC7325944

[42] Netea MG Balkwill F Chonchol M Cominelli F Donath MY Giamarellos-Bourboulis EJ. A guiding map for inflammation. Nat Immunol. (2017) 18:826–31. 10.1038/ni.3790 28722720 PMC5939996

[43] Liu L Wang W Wang Y Hou J Ying W Hui X. A Chinese DADA2 patient: report of two novel mutations and successful HSCT. Immunogenetics. (2019) 71:299–305. 10.1007/s00251-018-01101-w 30610243

[44] Moens L Hershfield M Arts K Aksentijevich I Meyts I. Human adenosine deaminase 2 deficiency: a multi-faceted inborn error of immunity. Immunol Rev. (2019) 287:62–72. 10.1111/imr.12722 30565235

